# Presumed Virus-Induced Punctal Occlusion

**DOI:** 10.1155/2014/809851

**Published:** 2014-11-13

**Authors:** Michael Yulish, Joseph Pikkel

**Affiliations:** ^1^Department of Ophthalmology, Ziv Medical Center, Safed, Israel; ^2^Bar-Ilan Faculty of Medicine, Safed, Israel

## Abstract

*Purpose.* To investigate viral infection as a cause of punctal stenosis in individuals without any ocular or systemic risk factors. *Methods.* The study group comprised patients with no known cause for punctal occlusion who underwent surgery at one medical center during a one-year period. Excised tissue was subjected to histological examination, PCR, and nested PCR testing for common viruses (adenovirus, influenza A and B, enterovirus, varicella-zoster, CMV, herpes simplex types 1 and 2, Epstein-Barr virus, and parainfluenza type 1). *Results.* All nine patients identified were female, 20–38 years of age. The three-snip-procedure resolved tearing in eight of them. All excised samples showed chronic mononuclear inflammation compatible with viral infection or with viral infection immune inflammatory reaction. PCR testing was negative for all the viruses examined; however, nested PCR was positive in three patients. *Conclusion.* This study supports the proposition that punctal occlusion in young healthy females may be due to viral infection.

## 1. Introduction

Punctal disorders are usually secondary to inflammation, infection, disuse, trauma, or medications. The punctum can be totally obliterated or stenotic. In herpes keratoconjunctivitis, the punctum and canaliculus may become stenotic [1]. In cases of longstanding ectropion or punctal ectropion alone, the punctal orifice may become stenotic or obliterated. The punctum may be occluded in systemic and ocular chronic graft-versus-host disease [2] and secondary to the usage of antineoplastic agents such as docetaxel (taxotere), as well as in patients with metastatic breast cancer [3] or chronic blepharitis [4]. Punctal or canaliculus occlusion may be associated with the use of certain topical ocular medications, including pilocarpine, phenylephrine hydrochloride, timolol maleate, indomethacin, and others, with a duration of exposure ranging from 3 weeks to 20 years [5]. In this study we examined the phenomenon of punctal occlusion or stenosis in young healthy patients without any ocular or systemic risk factors. We investigated the possibility that viral infections may be causal agents of chronic inflammation that results in punctual stenosis.

## 2. Patients and Methods

Patients suffering from epiphora who were operated due to unilateral or bilateral punctal occlusion at our ophthalmology clinic during the period from May 2011 to June 2012 were included in this study. Exclusion criteria were as follows: below 18 years of age, chronic eye disease (cataract, glaucoma, uveitis, blepharitis, allergic conjunctivitis, and dry-eye syndrome), chronic use of a topical ocular medication, and the presence of a systemic disorder (connective tissue disease, diabetes mellitus, and oncologic disorder). Inclusion criteria were two months to two years of tearing, positive FDDT (fluorescein dye disappearance test), free irrigation of nasolacrimal duct, punctal occlusion, or stenosis. All patients underwent a thorough ophthalmic evaluation including anamnesis of the current epiphora and medical history, full ophthalmic examination, and photographs of the lower canaliculus of both eyes. Punctal occlusion was graded according to the system suggested by Kashkouli et al. [6] (Table 1). All patients underwent a three-snip-procedure of the lower punctal occlusion, by the same surgeon (MY).

Excised tissue was subjected to histological examination and PCR testing for common viruses (adenovirus, influenza A, B, enterovirus, varicella-zoster, CMV, herpes simplex types 1 and 2, Epstein-Barr virus, and parainfluenza type 1) that are known to be the main conjunctival viral agents in the general population served by our medical center. When PCR testing results were negative a nested PCR examination for these viruses was performed (by the RAMBAM Medical Center laboratories, Haifa, Israel) using primers that were specific for each of the viruses that were mentioned above. The multiplex PCR step provided mass amplification using these common primers, which increased markedly the sensitivity of the test.

For sample processing we used the Chelex 100 (resin) because it was proven to be with high sensitivity and technically easy to perform in previous studies. 50 *μ*L of processed sediment was added to 200 *μ*L of 5% (wt/vol) Chelex 100; the mixture was then incubated at 55°C for 15 min, vortexed, boiled for 8 min, and spun for 2 min at 14,000 ×g. As primers for adenovirus, for example, we used a set which included 2 primers, (Designations: hexAA1885 and hexAA1913). For other viruses we used primers that were mentioned in previous studies as effective. The sequences of primers and names are described in Table 4. Amplification was done by adding a 10 *μ*L of the column-extracted sample to 40 *μ*L of master mix (Cloneal D 1X PCR Master Mix). Amplification in a thermocycler (Perkin-Elmer 9600) consisted of an initial round at 94°C for 7 min, 55°C for 1 min, and 72°C for 1.5 min, followed by 40 cycles at 94°C for 1 min, 55°C for 1 min, and 72°C for 1.5 min. Detection was done by separating the products using electrophoresis in a 4% (wt/vol) NuSieve agarose gel, stained with ethidium bromide, and photographed with UV trans illumination.

Demographic data, medical history, clinical findings, and surgical outcomes of all participants were collected and documented. Demographic data of the patients who suffered from punctual occlusion was collected as well (Table 3).

## 3. Results

Out of 83 patients that were operated because of punctual occlusion, nine patients were identified, all female. Their clinical characteristics are presented in Table 2. The mean age was 26.9 ± 5.8 years, and the range 20–38 years. The duration of symptoms prior to the three-snip procedure ranged from two to 23 months. Four of the patients remembered experiencing some degree of conjunctivitis before the beginning of tearing. For eight patients the operation resolved the tearing. One patient (number 2, Table 2) began to suffer from tearing due to common canaliculi occlusion eight months after the operation.

**Figure 1:**
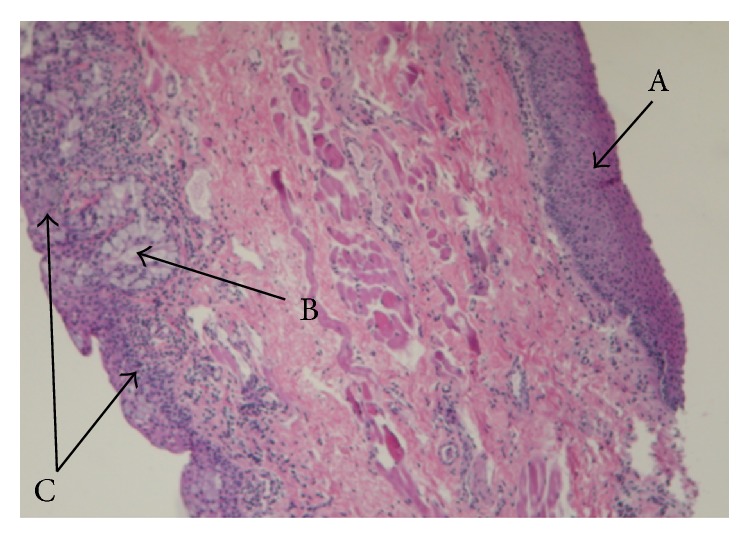
Histopathology of excised tissue. A: squamous epithelium of eyelid skin (epidermis) with no inflammation, B: goblet cells, and C: lichenoid lymphocytic inflammatory reaction in the conjunctival side of eyelid.

**Figure 2:**
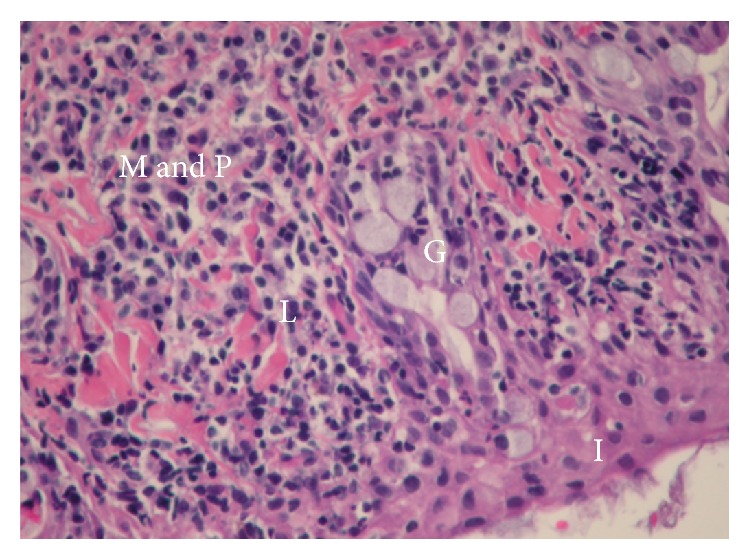
Chronic inflammatory reaction. G: goblet cells, I: infiltration of inflammatory cells to the epithelium, L: lymphocytes, and M & P: mononuclear and polymorphonuclear cells.

**Figure 3:**
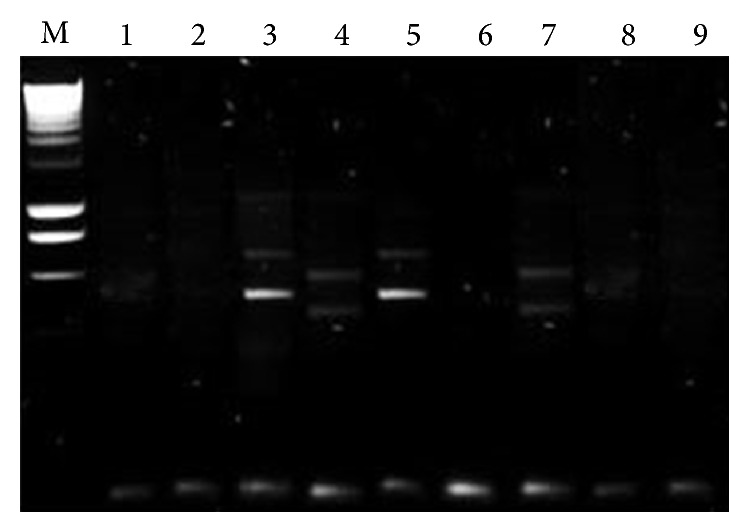
PCR results for adenovirus. Column M control. Columns 3 and 5 positive. Columns 1, 2, 6, and 9 negative. Columns 4 and 7 atypical.

All the samples showed chronic mononuclear inflammation compatible with viral infection or with viral infection immune inflammatory reaction (Figures 1 and 2). PCR testing was negative for all the viruses examined; however, nested PCR showed evidence of prior viral infection in three patients (two due to adenovirus and one due to influenza). Results of the nested PCR are shown in Figure 3. For these patients, the duration of symptoms of epiphora prior to surgery was 2-3 months, compared to 5 or more months for all the patients who tested negative.

## 4. Discussion

Punctal occlusion is a common condition, usually secondary to inflammation, infection, disuse, trauma, or the use of certain medications. In this study we examined the phenomenon of punctal occlusion or stenosis in patients without any ocular or systemic risk factors. The prevalence of punctal occlusion in such population is unknown, though it apparently comprises only a small fraction of all those with punctal occlusion. In our study we detected 92 (36%) patients suffering from punctual stenosis out of 254 patients that were referred to our oculoplastic service. 9 Patients out of 92 (9.8%) had punctual occlusion from an unknown cause. While Bukhari [7] detected punctal stenosis in 54.2% (370/682) of the patients who attended a general eye clinic for routine checkups, the cause was unknown in only 1.6% (6 patients).

We suspected a viral infection targeting the lacrimal punctum and canaliculi and causing chronic inflammation and punctal stenosis in our patients. One viral pathogen known to cause punctal occlusion is the double-stranded DNA virus,* Molluscum contagiosum* [8]. In the current study, PCR testing for common viruses in the excised tissue showed negative results in all samples; however, nested PCR showed evidence of prior viral infection in three patients. A possible explanation for the negative PCR results in six patients is that the causative viral infection agent was not tested (we examined only the most common conjunctival viral infection agents in this specific population). Another possibility is that the time elapsed between infection and excised tissue for PCR testing was too long for identification of viral agents. Indeed, the three patients, who were diagnosed as having a prior viral infection, had epiphora for a shorter period than did those for whom no viral agent was detected. It is likely that after a few months the causative virus no longer remains in the punctal tissue. While operating and testing excised tissue immediately after tearing seems advisable for the diagnosis of infective pathogens, it is not always possible. Another cause of chronic inflammation could be an unknown autoimmune process that targets punctal tissue.

Though our study consisted of a relatively small number of patients, we find that the possible link between viral infections and punctal occlusion is of importance and should be taken into consideration when treating these patients. Such a link was not, as far as we know, detected previously by PCR and has not been reported in the literature.

The 89% (8/9) success rate of the three-snip procedure observed in the current study is similar to the 92% (49/53) reported by Ceasar and McNab [9]. In our study and in theirs, successful outcome was defined as a subjective improvement in symptoms, specifically in epiphora. In a large retrospective study, anatomical success was achieved for 91% of patients who underwent the two-snip procedure and 94% of those who underwent the three-snip procedure (*P* = 0.7). However, of those achieving anatomical success, functional success was achieved in 71.4% undergoing the two-snip procedure, compared to 62.5% undergoing the three-snip procedure, *P* = 0.03 [10].

Lower punctal occlusion was the procedure performed in all patients in the current study. In a study that compared the effectiveness of lower and upper punctal occlusion for treating dry eye, no significant differences were observed in improvement of symptoms, corneal fluorescein staining intensity, and tear breakup time [11].

All nine of the patients in the current study were women. 75 (81.5%) out of the 92 patients that suffered from punctual occlusion were female. We do not know if punctal occlusion presents more in women or if women are more motivated to seek treatment. Gonnering and Kronish [8] reported women to comprise 74% of their patients with punctal stenosis who underwent the three-snip procedure. However, Kashkouli et al. [6] did not find any gender predilection among her patients with punctal stenosis. In a recently conducted population-based study, Viso et al. [12] reported prevalence of external punctal stenosis in 13.8% of men and 19.4% of women. None of these studies reported sex ratios for young and healthy individuals, as in our population.

## 5. Conclusion

This study supports viral infection and subsequent inflammation as a cause of punctal occlusion in healthy individuals. Nested PCR tissue testing demonstrated evidence of prior viral infection in individuals with a 2-3-month duration of epiphora.

## Figures and Tables

**Table 1:** Punctal occlusion grading^*^.

Grade	Clinical findings
0	No papilla and punctum
1	Papilla is covered by exudative membrane, true membrane, or fibrosis.
2	Punctum is less than normal size but recognizable
3	Normal punctum size
4	Punctum size less than 2 mm
5	Punctum size larger than or equal to 2 mm

^*^According to: M.B. Kashkouli et al. [6].

**Table 2:** Clinical characteristics of the study population.

Patient number	Age (years)	Duration of epiphora (months)	Punctum occlusion grade^*^	Nested PCR	PCR	Final outcome	Histological findings
1	36	6	0	Negative	Negative	No epiphora	Chronic mononuclear inflammation
2	28	23	2	Negative	Negative	Recurrent epiphora	Chronic mononuclear inflammation
3	22	2	1	Positive adenovirus	Negative	No epiphora	Chronic mononuclear inflammation
4	33	13	0	Negative	Negative	No epiphora	Chronic mononuclear inflammation
5	38	3	1	Positive *Haemophilus influenza *	Negative	No epiphora	Chronic mononuclear inflammation
6	20	12	1	Negative	Negative	No epiphora	Chronic mononuclear inflammation
7	25	5	0	Negative	Negative	No epiphora	Chronic mononuclear inflammation
8	29	3	1	Positive Adenovirus	Negative	No epiphora	Chronic mononuclear inflammation
9	30	14	0	Negative	Negative	No epiphora	Chronic mononuclear inflammation

^*^See Table 1.

**Table 3:** Demographic data.

Total number of oculoplastic patients	254
Patients suffering from punctual stenosis	92 (17 male/75 female)
Patients operated for punctal stenosis	83 (15 male/67 female)
Patients operated—punctal stenosis of unknown cause	9 (all female)

**Table 4:** Sequences of primers.

Virus	Sequence of primer
Adenovirus	5′-GCCGCAGTGGTCTTACATGCACATC-3′
	5′-CAGCACGCCGCGGATGTTCAAAGT-3′

*Haemophilus* *influenza *	5′-CTACTCATTGCAAGCATTGC-3′
	5′-GAATATGACCTGATCTTTCTG-3′
	5′-AGTGCACTATCCTGTTACAC-3′

Enterovirus	5′-GGCCCCTGAATGCGGCTAAT-3′
	5′-CAATTGTCACCATAAGCAGCCA-3′

Varicella-zoster	5′-GACAATATCATATACATG-3′

CMV	5′-TCAATCATGCGTTTGAAGAGGTA-3′
	5′-ACCACCGCACTGAGGAATGTCAG-3′

Herpes simplex	5′-TGCTCCTACAACAAGTC-3′

EBV	5′-TACAGGACCTGGAAATGGCC-3′
	5′-TCTTTGAGGTCCACTGCCG-3′

Parainfluenza	5′-ATTATGCAAACAAAACGTTCG-3′
	5′-GAAGAGTAAAACTAATTGCACAC-3′
	5′-GCAAGCACAACAAGTGCAGCTAA-3′
	5′-GCCGCCTTATCTAAACTTTCATCG-3′
